# A candidate DNA vaccine encoding a fusion protein of porcine complement C3d-P28 and ORF2 of porcine circovirus type 2 induces cross-protective immunity against PCV2b and PCV2d in pigs

**DOI:** 10.1186/s12985-019-1156-2

**Published:** 2019-05-02

**Authors:** Zhumei Hou, Honghua Wang, Yanni Feng, Qingwang Li, Junwei Li

**Affiliations:** 1 College of Animal Science and Technology, Northwest A&F University, Yangling, 712100 China; 2 College of Marine Science and Engineering, Qingdao Agricultural University, Qingdao, 266109 China; 3 Qingdao Vland Biotech Group Co.Ltd, Qingdao, 266061 China; 4 College of Veterinary Medicine, Qingdao Agricultural University, Qingdao, 266109 China

**Keywords:** DNA vaccine, C3d-P28, ORF2, PCV2b, PCV2d

## Abstract

**Background:**

Porcine circovirus type 2 (PCV2) is an economically important viral pathogen for swine industry worldwide. However, current PCV2 vaccines provide incomplete protection against the PCV2d, which has recently emerged as the predominant pathogenic form of PCV2.

**Methods:**

To develop a novel DNA vaccine with high efficacy against PCV2d virus, we fused the ORF2 of PCV2d to three copies of the minimum-binding domain of the complement C3 cascade terminal component, C3d-P28. Expression of ORF2 alone (pVO) or fused C3d-P28 (pVOC3) were verified by immunofluorescent assay. Vaccine efficacy was tested by measured the DNA copy and T and B cell immune response.

**Results:**

Vaccination with pVOC3 reduced the levels of PCV2 genomic DNA after pigs were infected with either PCV2b or PCV2d genotypes, produced potent antibodies against PCV2, and stimulated PCV2-specific interferon-γ secreting cells.

**Conclusion:**

Results suggested pVOC3 would be a safe and effective DNA vaccine to confer cross-protection against both PCV2b and PCV2d genotypes in pigs.

## Background

Porcine circovirus type 2 (PCV2) is a small, non-enveloped, circular, single-stranded DNA virus that belongs to the Circoviridae family [1]. As the etiological agent of postweaning multisystemic wasting syndrome (PMWS) and other PCV-associated diseases (PCVADs), PCV2 is one of the most economically important viral pathogens in the world-wide pig population [2]. There are four open reading frames (ORF) in genome of PCV2, ORF1 encoding a replication-associated protein, ORF2 encoding the major capsid protein, ORF3 coding for an apoptotic protein and ORF4 coding a apoptosis inhibitor respectively [3–5]. So far, there are five genotypes of PCV2, PCV2a, PCV2b, PCV2c, PCV2d, and PCV2e, have been identified, but PCV2c and PCV2e are relatively fewer descripted than other three genotypes. [6]. PCV2a was the predominant strain in global pig herds before 2003, then a shift from PCV2a to PCV2b occurred worldwidely [7]. However, PCV2d has been replacing PCV2a and PCV2b to become the predominant strain in pig populations since 2014 [6].

Vaccine is the most valuable tool in prevention of infectious diseases [8]. However, currently available commercial PCV2 vaccines, such as inactivated and subunit PCV2 vaccines are based on the PCV2a or PCV2b subtypes, and their efficacy against PCV2d is not clear. Some studies have demonstrated that the commercial PCV2a-based vaccines can significantly reduce PCV2b and PCV2d transmission under experimental conditions [1], but other studies suggest that PCV2 vaccines based on genotype 2b may be more effective than 2a-based vaccines at protecting against the PCV2d genotype [7]. Under the evolution pressure, PCV2 a mutated constantly and evidence suggests that the genetic gap between PCV2 vaccines and viruses is increasing [9, 10]. The incomplete protection against PCV2d infection by these vaccines may explain the global rise in PCV2d [11]. Thus, it is important to develop new approaches for vaccination to provide sufficient immune protection against the clinically dominant PCV2d.

Though DNA vaccines offer safety, genetic stability, ease of production, and induction of both humoral and cell-mediated immune responses [12], they elicit a slower rise in antibodies than protein or inactivated viral vaccines due to their poor antigenicity [13]. Thus, an ongoing area of research is focused on enhancing the immunological effect of DNA vaccines. Recent studies have demonstrated that a PCV2 ORF2 DNA vaccine can protect pigs against the PCV2b and that the response can be improved by administration of chemical adjuvants [14]. However, chemical adjuvants may be associated with toxicity, therefore approaches have been sought for increasing vaccine immunogenicity without excessive inflammation [15]. One approach to improve the response to DNA vaccines involves the use of molecular adjuvants [16]. The terminal degradation product (C3d) of mammalian complement component C3 has been used as a molecular adjuvant for DNA vaccines on the basis of its role in modulating the adaptive immune response through its interaction with complement receptor type 2 (CR2) on B cells [17]. Immunization of mice with hen-egg lysozyme (HEL) coupled with three C3d molecules greatly reduced the activation threshold of B cells, increased the immunogenicity of HEL by 1000-fold, which leads to stronger immune responses than that achieved with complete Freund’s adjuvant (CFA) [18]. Coupling multiple copies of C3d molecules or its minimum-binding domain C3d-P28 to target immunogens also has been shown to greatly enhance their specific response [19–21].

In this study, we constructed a recombinant plasmid that expresses three copies of C3d-P28 and PVC2d ORF2 (pVAX1-ORF2-C3d-P28.3). The ability of this DNA vaccine to elicit the humoral and cellular immune responses was investigated in piglets to protect pigs against both the PCV2b and PCV2d subtypes.

## Materials and methods

### Cells and viruses

PK-15 cells were grown and maintained in Dulbecco’s modified eagle medium (DMEM; Invitrogen) supplemented with 10% fetal calf serum and maintained in a 37 °C humidified chamber with 5% CO_2_. PCV2 strains LN-3 (PCV2b, MH920568) and HeB-1 (PCV2d, MH920550) were propagated and titrated in PK-15 cells.

### Plasmid construction

Eukaryotic expression plasmid pVAX1 was purchased from Invitrogen (USA). The full length ORF2 gene of PCV2 HeB-1 strain was amplified by using the primers 5′-ATCGCTAGCGCCGCCACCATGACGTATCCAA-3′ and 5′-CCCAAGCTTTCACTTAGGGTTAAGT-3′, cut with Nhe I/Hind III and ligated into pVAX 1 yielding pVAX1-ORF2. The ORF2-C3d-P28.3 fusion protein was designed by cloning three tandem repeats of the porcine homologue of C3d-P28 (HM026945.1) in frame at the 3′ end of the ORF2 gene. Linkers composed of two repeats of four glycines and a serine [(G4S)2] were fused at the junctures of ORF2 and C3d-P28 and between each C3d-P28 repeat. The ORF2-C3d-P28.3 gene with Nhe I/Hind III in 5′ and 3′ ends was synthesized commercially (Takara, Japan) and was ligated into pVAX1 yielding pVAX1-ORF2-C3d-P28.3.

*Escherichia coli* strain DH5a was used as the host for all plasmids. Plasmids were purified from cultures of *E. coli* using an EndoFree Plasmid Mega kit (QIAGEN). Plasmids were verified by appropriate restriction enzyme digestion and gel electrophoresis. The purity of DNA preparations was verified by optical density reading at 260 and 280 nm.

### Transfection and expression analysis

PK-15 cells (5 × 10^5^ per transfection) were transfected with 2 μg of plasmid DNA by using LipofectAMINE 2000 (Invitrogen, USA) according to the manufacturer’s instructions. After 48 h, the cells were gently washed with PBS (pH 7.4) and fixed in 4% paraformaldehyde for 15 min. The infected cells were washed again and incubated with 2 ml of a 1:100 dilution of primary antibody (rabbit polyclonal anti-PCV2) at 37 °C for 1 h. After being washed, goat anti rabbit IgG conjugated with FITC (1:5000) was added, and the cells were incubated for 60 min at 37 °C. After 3 washes, fluorescence in the cells was visualized under a fluorescence microscope.

### Experimental use of animals

Thirty five male piglets aged 3 weeks old that tested negative for PCV2 antigen and antibody were selected randomly for the experimental groups. The animal study proposal was approved by the Institutional Animal Care and Use Committee (IACUC) of the Shandong province. All animal care and experiments were carried out in accordance with the Regulations for the Administration of Affairs Concerning Experimental Animals approved by the State Council of People’s Republic of China.

The piglets were vaccinated intramuscularly in the right-hand side of the neck (500 μg each). Three weeks later, piglets were individually boosted. Five weeks after primary immunization (56 days of age), piglets were challenged intranasally with 2.0 mL of the wild-type strain LN-3 (PCV2b) or HeB-1 (PCV2d) strain at a titer of 10^5.5^ tissue culture infective dose (TCID_50_)/ml. Piglets were monitored for 21 days post-challenge and then were euthanized (shown in Table 1).

**Table 1:** Experimental design

Group	Immunized recombinant plasmid	Dose	Challenge isolate	Challenge dose (TCID_50_/ml)
pV/PCV2b	pVAX1	400 μg/ml	PCV2b	2 × 10^5.5^
pV/PCV2d	pVAX1	400 μg/ml	PCV2d	2 × 10^5.5^
pVO/PCV2b	pVAX1-ORF2	400 μg/ml	PCV2b	2 × 10^5.5^
pVO/PCV2d	pVAX1-ORF2	400 μg/ml	PCV2d	2 × 10^5.5^
pVOC3/PCV2b	pVAX1-ORF2-C3d-P28.3	400 μg/ml	PCV2b	2 × 10^5.5^
pVOC3/PCV2d	pVAX1-ORF2-C3d-P28.3	400 μg/ml	PCV2d	2 × 10^5.5^
Negative group	PBS	1 ml	/	/

The rectal temperature and clinical examination data of piglets were recorded daily. Body weight was measured weekly and their relative daily weight gain (ADWG) was determined. Blood samples were collected on a weekly basis for PCV2 antibodies, quantitative PCR analysis of the genomic copies of PCV2 and PCV2-specific IFN-γ-SC.

### PCV2 measurement by serology

Serum samples were tested with the PCV2 ELISA kit (J.B.T., Korea). Samples were considered positive if the calculated sample to positive (S/P) ratio was ≥0.4. Viral DNA in serum samples was also extracted using QIAamp DNA Mini Kit (Qiagen, Hilden, Germany) according to the manufacturer’s instructions and subjected to digital PCR for the detection of PCV2 [8]. Briefly, primers, SYBR Green I Mix (TaKaRa), DNA templates, and ddH2O were mixed in PCR tube up to 25 lL. then PCR amplification was performed as 95 °C for 10 min, followed by 30 cycles of amplification at 95 °C for 10 s, and 62 °C for 10 s. Melt curve analysis was performed at 95 °C for 2 min, 60 °C for 20 s, and 95 °C. Primers are available upon being requested.

### Microscopic lesions and immunohistochemistry (IHC)

Microscopic lesions and PCV2 immunohistochemistry of lymph nodes were evaluated as previously described [22]. Briefly, lymphoid nodes were collected after euthanization and were assigned histopathological lesion scores ranging from 0 (none) to 3 (severe) by two veterinary pathologists blinded to treatment status. The PCV2 antigens of lymphoid nodes were detect by PCV2 IHC, and positive signals were normalized using the NIH Image J 1.45 s Program (http://imagej.nih.gov/ij/download.html).

### Determination of IFN-γ-SC specific to PCV2

An Enzyme-linked immunospot (ELISPOT) was used to determine frequencies of PCV2-specific IFN-**γ**-SC in isolated peripheral blood mononuclear cells (PBMC) as previously described [23].

### Statistical analysis

S/P ratios, PCV2 DNA and PCV2-specific IFN-**γ**-SC were analyzed by using two-way analysis of variance (ANOVA), while lymphoid lesion score and PCV2-antigen score were analyzed by using one-way analysis of variance (ANOVA). All data were performed using GraphPad Prism v7.0 (GraphPad Software, La Jolla, CA, United States) to calculate statistical significance (*P* < 0.05, significant difference).

## Results

### Construction and expression of recombinant vaccine vectors

**Fig. 1:**
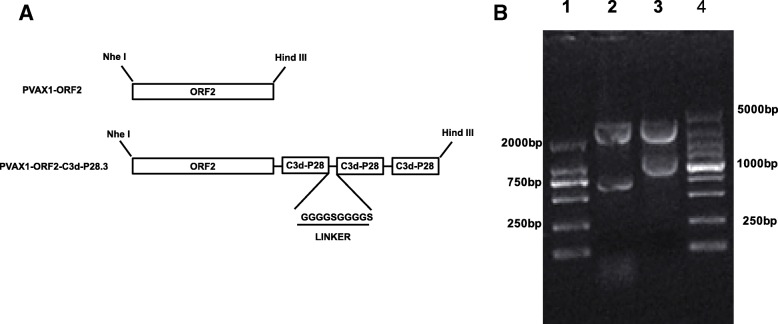
Construction of the plasmids PVAX1-ORF2 and PVAX1-ORF2-C3d-P28.3 (**a**) Top: the ORF2 construct that was used as a vaccine insert; Bottom: the ORF2-C3d-P28.3 construct that was used as a vaccine insert. Linkers composed of two repeats of four glycines and a serine [(G4S)2] were fused at the junctures of ORF2 and C3d and between each C3d repeat. **b** Restriction digest of PVAX1-ORF2 and PVAX1-ORF2-C3d-P28.3. Lane 1: DL2000 marker; lane 2: PVAX1-ORF2 digested with Nhe I/Hind III; lane 3: PVAX1-ORF2-C3d-P28.3 digested with Nhe I/Hind III; lane 4: DL5000 marker

To test the adjuvant effect of combining ORF2 with porcine C3d-P28 complement, two plasmids were engineered in pVAX1, generating the vaccine pVAX1-ORF2 and the chimeric vaccine pVAX1-ORF2-C3d-P28.3 (Fig. 1a). The pVAX1-ORF2 vaccine contains the entire ORF2 gene coding region of PCV2d, and the pVAX1-ORF2-C3d-P28 vaccine contains the fusion protein gene of ORF2 gene and three tandem repeats of the porcine C3d-P28 gene. Two repeats of (G_4_S)_2_ were fused at the juncture of ORF2 and porcine C3d-P28 and between each C3d-P28 repeat. Restriction digests for the resultant plasmids produced fragments of the expected sizes. (Fig. 1b).

**Fig. 2:**
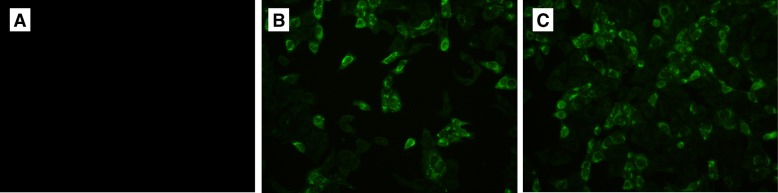
In vitro expression of DNA vaccine constructs. DNA vaccine constructs were transfected into PK-15 cells and then stained with rabbit polyclonal anti-Cap of PCV2 followed by FITC-labelled goat anti-rabbit antibody. **a** negative control; (**b**) pVAX1-ORF2; (**c**) pVAX1-ORF2-C3d-P28.3

To verify the expression of ORF2 and ORF2-C3d-P28.3 proteins, PK-15 cells were transiently transfected with the plasmids and then were subjected to immunofluorescent staining. The results indicate that both the pVAX1-ORF2 and chimeric pVAX1-ORF2-C3d-P28.3 vaccines were expressed in PK-15 cells (Fig. 2).

### PCV2-specific antibodies are produced in pigs at high levels in response to pVOC3 vaccine

To evaluate the protective effect of the vaccines, the pigs were divided into seven groups (*n* = 5 pigs per group; Table 1). The groups were unvaccinated (negative group) or were vaccinated with pVAX1, pVAX1-ORF2, or pVAX1-ORF2-C3d-O28.3 and then were boosted with the same vaccines three weeks later. At five weeks after primary immunization, piglets were challenged with PCV2b or PCV2d viruses and then had monitored for 21 days. None of the pigs developed clinical signs consistent with PCVAD throughout the study, and no difference in weight gain was observed in each group. Since after vaccination, the average daily weight gain (ADWG) was 0.21 to 0.23 kg/day, and after challenge to the endpoint of experiment, the ADWG ranged from 0.24 to 0.27 kg/day, with no significant differences between the groups (Table 2). Furthermore, no fever was observed in all pigs.

**Table 2:** Average Daily Weight Gain (ADWG) of experimental pigs from vaccination day to end of experiment

Group	ADWG	
	Vaccination to challenge (days 0 to 35)	Challenge to end of experiment (days 35–56)
pV/PCV2b	0.22 ± 0.11	0.24 ± 0.08
pV/PCV2d	0.23 ± 0.09	0.25 ± 0.13
pVO/PCV2b	0.22 ± 0.13	0.24 ± 0.12
pVO/PCV2d	0.22 ± 0.07	0.26 ± 0.07
pVOC3/PCV2b	0.21 ± 0.10	0.27 ± 0.09
pVOC3/PCV2d	0.23 ± 0.12	0.25 ± 0.08
Negative group	0.22 ± 0.09	0.26 ± 0.10

**Fig. 3:**
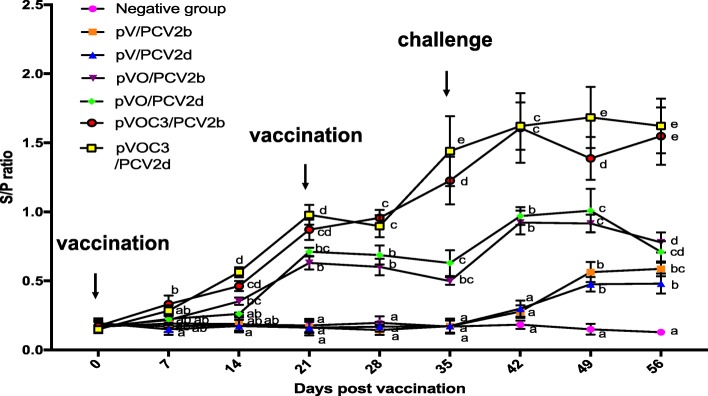
Production of PCV2 antibodies in pigs after vaccination and infection with PCV2b or PCV2d viruses. Pigs in each of seven groups (*n* = 5 per group) were vaccinated on day 0, boosted on day 21, and infected with PCV2 on day 35. The mean sample-to-positive (S/P) ratio of each group at different days post vaccination were measured weekly by PCV2 ELISA as an indication of the host response. Different superscripts (^a,b,c,d,e^) indicate significant differences among groups. (*p* < 0.05)

To assess immune response to the vaccines, antibody titers in the serum were measured. At 0 and 7 days post vaccination (DPV), all pigs were negative for PCV2-specific antibodies. Positive PCV2 antibodies were detected in the serum of pigs of each vaccinated with DNA vaccine carrying ORF2 (pVO/PCV2b, pVO/PCV2d, pVOC3/PCV2b, and pVOC3/PCV2d) starting at day 14 DPV (Fig. 3). Pigs from the pVOC3 groups had significantly (*p* < 0.05) higher S/P ratios than those of the pVO groups from 14 to 56 DPV. The pigs in pVAX1/PCV2b and pVAX1/PCV2d groups, which received an empty vector, sera-convertion at day 49 (14 days after PCV2 challenge). These results indicate that the pVAX1-ORF2 and pVAX1-ORF2-C3d-O28.3 are able to elicit antibody responses in pigs prior to PCV2 infection and that the humoral immune response stimulated by pVAX1-ORF2-C3d-28.3 was potent.

### pVOC3 vaccination reduces viremia upon infection with PCV2b or PCV2d

**Fig. 4:**
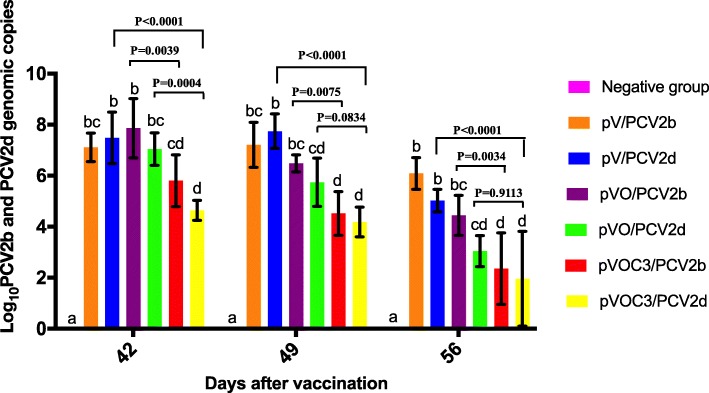
PCV2 DNA in sera after vaccination and infection of pigs with PCV2b or PCV2d. The PCV2b or PCV2d genomic copies in serum were measured at days 7, 14, and 21 after virus challenge and were expressed as the mean log_10_ quantities of 5 pigs per group. No DNA was detected prior to viral challenge. Different superscripts (^a,b,c,d^) indicate significant differences among groups. (*p* < 0.05)

To further evaluate whether the vaccines can protect pigs against infection with both PCV2b and PCV2d viruses, we assessed the levels of virus in the sera of infected pigs. PCV2 DNA was not detected in any of the serum samples from the 7 groups prior to challenge; however, viral DNA could be quantified after infection with PCV2b or PCV2d (Fig. 4). Among pigs immunized with the same vaccine, there were no significant differences of genomic copies for PCV2b and PCV2d (*P* > 0.05). Pigs from the pVOC3/PCV2b and pVOC3/PCV2d groups had significantly (*P* < 0.05) less PCV2 genomic copies in their sera compared with pigs from each of the other challenge groups at 14, and 21 DPC. Much significantly, the genomic copies of PCV2 in pVOC3/PCV2b and pVOC3/PCV2d groups decreased around 10^4^–10^5^ fold compared with empty vector groups at 21 DPC. These results suggest that the pVAX1-ORF2-C3d-O28.3 DNA vaccine is able to reduce the infection outcome either by PCV2b or PCV2d.

### pVOC3 vaccination reduces the levels of PCV2 antigen after infection with PCV2b or PCV2d

To further evaluate the efficacy of the pVAX1-ORF2-C3d-P28.3, we evaluated the lesions and the amount of PCV2 antigen in pigs after vaccination and virus challenge. The lymphoid lesion score was reduced after vaccination; however, the differences were not statistical significance (*P* > 0.05; Table 3, first row). The greatest reduction in the PCV2 antigen score, however, was observed in the pigs from the pVOC3/PCV2b and pVOC3/PCV2d groups, which had significantly (*P* < 0.05) lower antigen compared to the pV/PCV2b and pV/PCV2d groups. No lymphoid lesions or PCV2-antigen were detected in lymph nodes of pigs from the negative group (Table 3). These results support that pVAX1-ORF2-C3d-P28.3 reduces viral replication.

**Table 3:** The lymphoid lesion score and PCV2-antigen score of experimental pigs

	Negative group	pV/PCV2b	pVO/PCV2b	pVOC3/PCV2b	pV/PCV2d	pVO/PCV2d	pVOC3/PCV2d
Lymphoid lesion score	0.20 ± 0.45^a^	1.80 ± 0.84^bc^	1.20 ± 0.84 ^ac^	0.80 ± 0.84 ^ac^	2.20 ± 0.84 ^bc^	1.00 ± 1.00 ^ac^	0.60 ± 0.89 ^ac^
PCV2-antigen score	0 ^a^	18.80 ± 4.44 ^b^	8.20 ± 5.22 ^a^	5.40 ± 3.97 ^a^	20.6 ± 5.03 ^b^	7.60 ± 5.18^a,^	3.40 ± 3.58 ^a,^

Different superscripts (^a,b,c^) indicate significant differences among groups. (*p* < 0.05)

### Vaccination with pVOC3 increases the number of PCV2-specific interferon-γ secreting cells

**Fig. 5:**
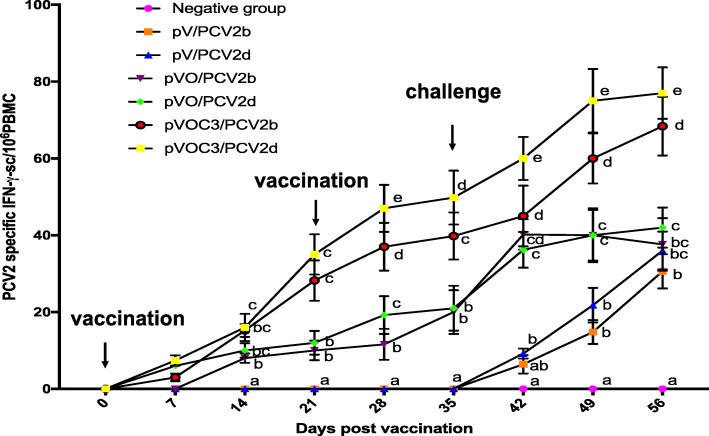
PCV2-specific cell-mediated immune responses to PCV2b and PCV2d are stimulated by vaccination. The numbers of PCV2-specific interferon-γ secreting cells (IFN-**γ**-SC)/10^6^ peripheral blood mononuclear cells (PBMC) were assessed weekly after vaccination (on day 0 and 21) and PCV2 infection (on day 35). Different superscripts (^a,b,c,d,e^) indicate significant differences among groups. (*p* < 0.05)

To further evaluate T cell immune response stimulated by our DNA vaccine candidates, we measured PCV2-specific interferon-**γ** secreting cells (IFN-**γ**-SC) for each experimental group (Fig. 5). Pigs from the pVO/PCV2b, pVO/PCV2d, pVOC3/PCV2b, and pVOC3/PCV2d groups had significantly (*P* < 0.05) higher numbers of PCV2-specific IFN-**γ**-SC compared to pigs from the other groups at 14, 21, 28, 35, 42, 49, and 56 DPV. Furthermore, pVOC3/PCV2b and pVOC3/PCV2d groups had significantly (*P* < 0.05) higher numbers of PCV2-specific IFN-**γ**-SC compared to pigs from the pVO/PCV2b and pVO/PCV2d groups at 21, 28, 35, 42, 49, and 56 DPV. No PCV2-specific IFN-**γ**-SC was detected in pigs from the negative group. Therefore, vaccination with, in particular, the pVAX1-ORF2-C3d-P28.3 DNA vaccines that has three copies of C3d, significantly stimulates PCV2-specific IFN-**γ**-SC, which may help to inhibit infection with both PCV2b and PCV2d.

## Discussion

The economic impact of PMWS has released with vaccination of piglets, age-adjusted diets and reduction of stock density [24, 25]. As emerging of PCV2d genotype, new formulation of PCV vaccine should be considered [26]. Based on the predominance of PCV2d, a current research focus has been on the development of a vaccine that can effectively prevent infection by PCV2d. In this study, we developed a DNA vaccine based on ORF2 of PCV2d, and demonstrated its efficacy against both PCV2b and PCV2d. As a novel approach to improving the immunogenicity of the vaccine, we fused ORF2 to three copies of the minimum-binding domain of the complement C3 cascade terminal component, C3d-P28. Our results suggest that vaccination with the ORF2-C3d fusion DNA vaccine elicited immune response superior to that stimulated by ORF2 DNA vaccine alone.

The superior efficacy of the C3d-P28-fused vaccine was demonstrated by several approaches. First, we evaluated the production of PCV2-specific antibodies. Our results showed that vaccination elicited the production of antibodies that preceded and superceded the levels elicited by virus exposure. We performed serological evaluation of the number of genomic copies and the amount of PVC2 antigen, which suggested that vaccination prior to infection decreases viremia. Finally, we demonstrated that vaccination increased the development of PCV2-specific IFN-**γ**-SC. Each of these approaches has been used to evaluate PCV2 vaccine efficacy in other studies [27, 28], and each of our findings are consistent with the protective efficacy of our vaccine.

Our vaccine design incorporates several advantageous features. First, our strategy involves the development of a DNA vaccine. Because of the favorable safety, ease of production and cost relative to other vaccination approaches, DNA vaccines have been used increasingly, particularly in the fish and poultry industries [29]. Second, the viral sequence was fused to C3d as a safe and controlled molecular adjuvant for increasing immunogenicity without the need for chemical adjuvants [16]. C3d fusion in DNA vaccination had been used to boost the immune response against a variety of animal viruses, including chicken viral Newcastle disease [30], bovine viral diarrhea virus [31], and swine influenza virus [32], however, this is the first study to assess C3d fusion to PCV2. Third, our vaccine used ORF2 of PVC2, which is highly conserved among the different genotypes [3]. Given the high mutation rate of the PCV2 [9], we hypothesized that the use of a conserved viral component might provide broad immunity, and this was evidenced by the immune response observed for the ORF2 vaccines against both PCV2b and PCV2d. Promising results have been recently demonstrated for DNA vaccination using a PCV2 ORF2 vaccine to restrain PCV2b, but C3d fusion was not investigated in the latter study and the effectiveness against PCV2d was not evaluated [14]. Therefore, our approach provides several advances over previous methods of PCV2 vaccination and is shown to target the predominant worldwide form of the virus, PCV2d. Future studies to assess the cost savings of this method and to establish the safety in larger pig populations should help to support the utility of PCV2/C3d vaccination for protecting pigs against PCV2d and other emerging genotypes of PCV2.

## Conclusion

In this study, we designed a DNA vaccine in which a gene segment of the complement cascade was fused to Cap gene of PCV2d as adjuvant. Our results demonstrate that vaccination with this DNA vaccine candidate in pigs significantly inhibited replication of PCV2b and PCV2d and induced potent humoral and T cell immune response. It indicates that this innovative DNA vaccine is possible confer broad and potential application in prevent and control PCV.

## Abbreviations

ADWG: Average daily weight gain
ANOVA: Analysis of variance
C3d-P28: Complement C3 cascade terminal component
CFA: Complete Freund’s adjuvant
DMEM: Dulbecco’s modified eagle medium
DPV: Days post vaccination
ELISPOT: Enzyme-linked immunospot
HEL: Hen-egg lysozyme
IFN-γ-SC: Interferon-γ secreting cells
IHC: Immunohistochemistry
ORF: Open reading frame
PBMC: Peripheral blood mononuclear cells
PCV2: Porcine circovirus type 2
PCVADs: PCV-associated diseases
PMWS: Postweaning multisystemic wasting syndrome
S/P: Sample to positive
TCID_50_: Tissue culture infective dose
